# The ribonucleoside AICAr induces differentiation of myeloid leukemia by activating the ATR/Chk1 via pyrimidine depletion

**DOI:** 10.1074/jbc.RA119.009396

**Published:** 2019-08-20

**Authors:** Vilma Dembitz, Barbara Tomic, Ivan Kodvanj, Julian A. Simon, Antonio Bedalov, Dora Visnjic

**Affiliations:** ‡ Department of Physiology and Croatian Institute for Brain Research, School of Medicine, University of Zagreb, 10 000 Zagreb, Croatia; § Clinical Research Division, Fred Hutchinson Cancer Research Center, Seattle, Washington 98109

**Keywords:** leukemia, cell cycle, cell differentiation, nucleotide, pyrimidine, DNA damage response, AMP-activated kinase (AMPK), AICAr, AML, Chk1

## Abstract

Metabolic pathways play important roles in proliferation and differentiation of malignant cells. 5-Aminoimidazole-4-carboxamide ribonucleoside (AICAr), a precursor in purine biosynthesis and a well-established activator of AMP-activated protein kinase (AMPK), induces widespread metabolic alterations and is commonly used for dissecting the role of metabolism in cancer. We have previously reported that AICAr promotes differentiation and inhibits proliferation of myeloid leukemia cells. Here, using metabolic assays, immunoblotting, flow cytometry analyses, and siRNA-mediated gene silencing in leukemia cell lines, we show that AICAr-mediated differentiation was independent of the known metabolic effects of AMPK, including glucose consumption, but instead depends on the activation of the DNA damage–associated enzyme checkpoint kinase 1 (Chk1) induced by pyrimidine depletion. LC/MS/MS metabolomics analysis revealed that AICAr increases orotate levels and decreases uridine monophosphate (UMP) levels, consistent with inhibition of UMP synthesis at a step downstream of dihydroorotate dehydrogenase (DHODH). AICAr and the DHODH inhibitor brequinar had similar effects on differentiation markers and S-phase arrest, and genetic or pharmacological Chk1 inactivation abrogated both of these effects. Our results delineate an AMPK-independent effect of AICAr on myeloid leukemia differentiation that involves perturbation of pyrimidine biosynthesis and activation of the DNA damage response network.

## Introduction

Lack of differentiation is a hallmark of cancer, making differentiation therapy a promising treatment strategy. The most successful example of this approach is all-*trans*-retinoic acid (ATRA)^2^-based therapy of acute promyelocytic leukemia (APL), a subtype of acute myeloid leukemia (AML) carrying a t(15,17) translocation. As the proposed mechanism of APL differentiation involves binding of ATRA to the fusion protein encoded by t(15,17) and its degradation, ATRA-based therapy is restricted to APL, whereas all other AML subtypes are treated by a combination of cytotoxic drugs (1). Several novel differentiation targets in AML and other cancers have been identified, including nucleotide biosynthesis pathways (2, 3), lysine-specific demethylases (4), and mutant isocitrate dehydrogenase (IDH) (5, 6). Identification of 2-hydroxyglutarate as an oncometabolite produced by mutant IDH1 and IDH2 enzymes (7) has invigorated interest in the role of intermediary metabolism in regulation of cell differentiation and growth.

AMP-activated protein kinase (AMPK) is a principal regulator of cellular metabolism in response to energy status of the cell. Activated AMPK facilitates catabolic pathways, like glycolysis and fatty acid oxidation, and decreases anabolic pathways, like protein and fatty acid syntheses, which makes AMPK an attractive target to modulate the metabolism. Two drugs that are commonly used to activate AMPK, the antidiabetic biguanide metformin and 5-aminoimidazole-4-carboxamide ribonucleoside (AICAr or acadesine), use different mechanisms to activate AMPK. Metformin employs a complex pathway that involves inhibition of mitochondrial oxidative phosphorylation. AICAr enters the cell through adenosine transporters and becomes phosphorylated by adenosine kinase into 5-aminoimidazole-4-carboxamide ribonucleotide (AICAR; also termed ZMP), which acts as an AMP mimetic. Endogenous AICAR is also an intracellular intermediate in the *de novo* purine biosynthesis that is known to accumulate in Lesch–Nyhan syndrome and other purine synthesis disorders (8). Even though both metformin and AICAr are commonly used as AMPK activators in studies related to metabolism and the insulin signaling pathway, an increasing number of studies have demonstrated that at least some of their effects are actually AMPK-independent (9–12).

Our previous study testing the effects of drugs that modulate the activity of AMPK revealed that AICAr induced the expression of differentiation markers in AML cell lines. In U937 cells, both AICAr and metformin induced time- and dose-dependent activation of AMPK, but AICAr-mediated effects on differentiation did not depend on the presence of AMPK. Moreover, no differentiation was induced by metformin, further suggesting that the pathway of differentiation involves mechanisms other than AMPK activation (13). The goal of this study was to define the metabolic pathways necessary for AICAr-mediated differentiation. Here, we show that AICAr-mediated effects depend on pyrimidine synthesis and the activity of checkpoint kinase 1 (Chk1) and that both AICAr-mediated arrest in S phase and the expression of differentiation markers can be abolished by the addition of uridine.

## Results

### AICAr-induced differentiation is independent of glycolysis

**Figure 1.**
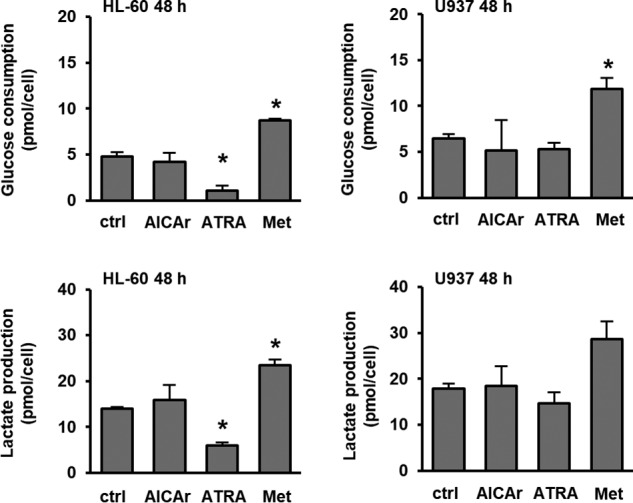
**AICAr has no effects on glucose consumption or lactate production in U937 cells.** HL-60 and U937 cells were treated with AICAr (0.5 mm), ATRA (1 μm), and metformin (*Met*) (15 mm) for 48 h, and the levels of glucose and lactate were measured in culture medium. Results are mean ± S.E. (*error bars*) of at least three independent experiments. *, *p* < 0.05 compared with control (*ctrl*).

To gain broad insight into metabolic effects of AICAr during leukemia differentiation, we analyzed its effect on glucose consumption and lactate production. In parallel, we examined metabolic effects of ATRA to determine whether the effects on metabolism are specific to AICAr or are generic metabolic adaptation during differentiation. Cells were incubated with ATRA at the concentration that has been previously described to decrease glucose consumption in HL-60 cells in parallel with an increase in the expression of differentiation markers (14). In addition, metformin was added as an AMPK agonist at a concentration that was previously shown to have no effects on differentiation in either myeloblastic HL-60 or monocytic U937 cells (13) but to induce a switch to glycolysis during apoptosis (15). As shown in Fig. 1, metformin increased and ATRA decreased glucose consumption and lactate production in both cell lines after 48 h of incubation. No significant effects on glucose consumption and lactate production were observed in the presence of AICAr. Thus, we conclude that AICAr-induced differentiation does not inhibit glycolysis, and the effect was specific to AICAr.

### AICAr-induced arrest in S phase and differentiation are abolished by nucleotides and uridine

**Figure 2.**
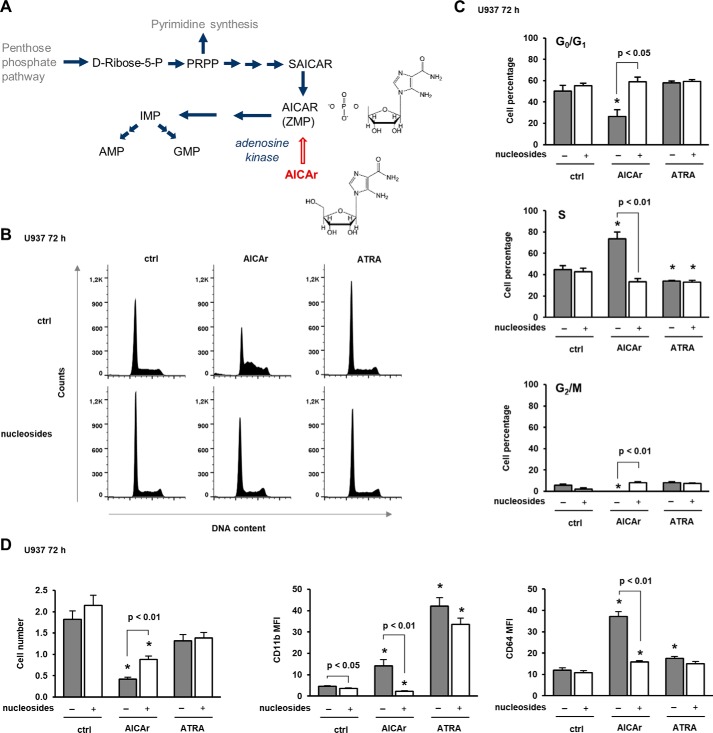
**Nucleosides abolish AICAr-mediated S-phase arrest and the increase in the expression of differentiation markers.** *A*, *de novo* purine synthesis pathway. *SAICAR*, succinyl-5-aminoimidazole-4-carboxamide-1-ribose-5′-phosphate. *B–D*, U937 cells were incubated with AICAr (0.5 mm), ATRA (1 μm), and nucleosides (1×) for 72 h. *B*, representative histograms of propidium-labeled cells analyzed by flow cytometry. *C*, percentage of cells in G_0_/G_1_, S, and G_2_/M phases of the cell cycle. *D*, the number of viable cells and the expression of differentiation markers were determined as described under “Experimental procedures.” Results are mean ± S.E. (*error bars*) of at least three independent experiments. *, *p* < 0.05 compared with control (*ctrl*).

Because AICAr participates in nucleotide biosynthesis (Fig. 2*A*) and perturbation of nucleotide pool is associated with S-phase delay (16), we next tested the effects of AICAr on the progression through the cell cycle. AICAr- and ATRA-treated cells were stained with propidium iodide, and the percentage of cells in the particular phase of the cell cycle was determined by flow cytometry. As shown in Fig. 2, *B* and *C*, two differentiation agents had opposite effects; AICAr caused an arrest in S phase, whereas ATRA significantly decreased the percentage of cells in S phase. Because S-phase arrest may be due to depletion of the nucleotide pool, we asked whether addition of nucleosides could alter the cellular response to AICAr. As shown in Fig. 2, *B* and *C*, the addition of the mixture of nucleosides (A, G, C, T, and U) abolished AICAr-mediated effects on the cell cycle. Additionally, the mixture of nucleosides significantly increased the number of viable cells upon treatment with AICAr and inhibited the AICAr-mediated increase in the expression of differentiation markers (Fig. 2*D*).

**Figure 3.**
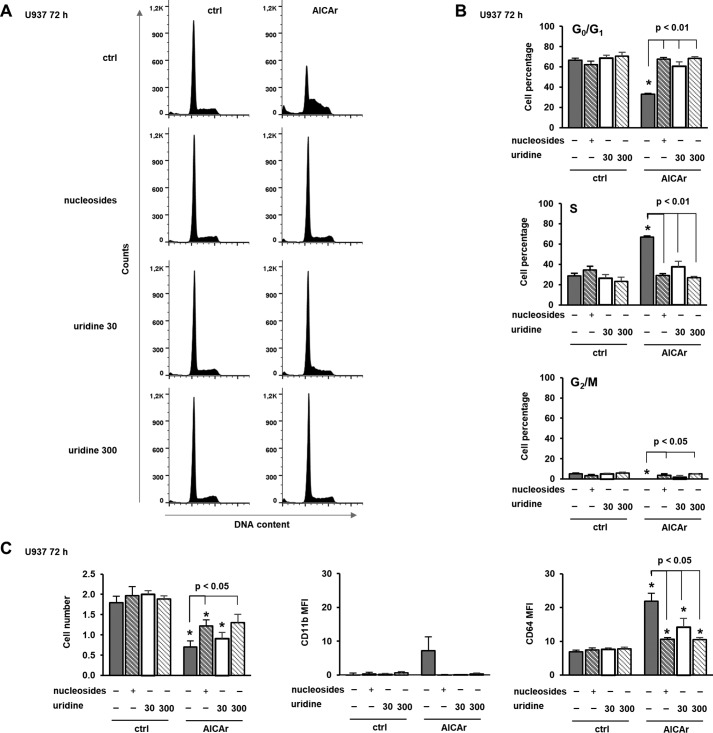
**Uridine dose-dependently prevents AICAr-mediated S-phase arrest and the increase in the expression of differentiation markers.** U937 cells were incubated with AICAr (0.5 mm), nucleosides (1×), and uridine (30 and 300 μm) for 72 h. *A*, representative histograms of propidium-labeled cells analyzed by flow cytometry. *B*, percentage of cells in G_0_/G_1_, S, and G_2_/M phases of the cell cycle. *C*, the number of viable cells and the expression of differentiation markers were determined as described under “Experimental procedures.” Results are mean ± S.E. (*error bars*) of at least three independent experiments. *, *p* < 0.05 compared with control (*ctrl*).

The abrogation of AICAr-mediated differentiation with nucleosides suggests that AICAr might interfere with their synthesis. In multiple myeloma cells, AICAr inhibits uridine monophosphate (UMP) synthesis, and the apoptotic effect of AICAr is prevented by the addition of uridine (17). Therefore, we asked whether the addition of uridine can likewise rescue the effects of AICAr on myeloid cells. As shown in Fig. 3, uridine dose-dependently prevented AICAr-mediated S-phase arrest and the increase in the expression of differentiation markers. We conclude that both AICAr-mediated arrest in S phase and differentiation are caused by a defect in pyrimidine synthesis.

### Low concentrations of AICAr and brequinar had synergistic effects on differentiation markers and S-phase arrest

**Figure 4.**
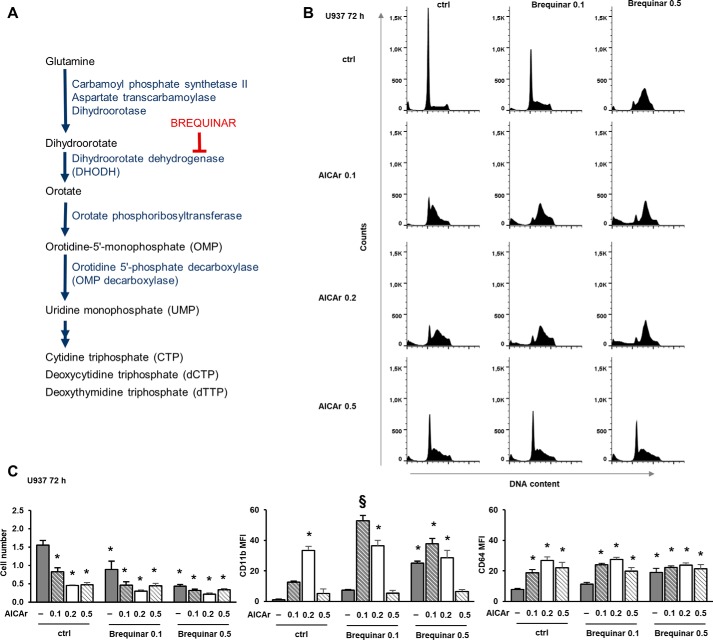
**Low concentrations of AICAr and brequinar have synergistic effects on the S-phase arrest and the expression of differentiation markers.** *A*, *de novo* pyrimidine synthesis pathway. *B* and *C*, U937 cells were incubated with AICAr (0.1, 0.2, or 0.5 mm), brequinar (0.1 or 0.5 μm), and their combinations for 72 h. *B*, representative histograms of propidium-labeled cells from three independent experiments analyzed by flow cytometry. *C*, the number of viable cells and the expression of differentiation markers were determined as described under “Experimental procedures.” Results are mean ± S.E. (*error bars*) of at least three independent experiments. *, *p* < 0.05 compared with control (*ctrl*). §, *p* < 0.05 compared with both agents alone.

A recent study provided a link between uridine and cell differentiation; the pharmacological inhibition of dihydroorotate dehydrogenase (DHODH), which catalyzes the fourth step in *de novo* pyrimidine synthesis, overcame differentiation blockade in acute myeloid leukemia cells. The DHODH inhibitor brequinar increased the expression of differentiation markers in U937 cells, but the effect on the cell cycle or DNA damage response was not tested (3). As shown in Fig. 4*A*, DHODH acts upstream of UMP synthesis, converting dihydroorotate (DHO) to orotate. Consequently, the inhibition of the enzyme activity increases an upstream metabolite (DHO) and depletes uridine metabolites; myeloid differentiation is due to the lack of uridine and not the accumulation of DHO (3). Our results suggest that AICAr exerts effects on the pyrimidine biosynthesis pathway and that both effects on the cell cycle and differentiation are due to the lack of uridine. To test the hypothesis that myeloid differentiation in response to AICAr shares the same mechanism of action as that induced by DHODH inhibitors, U937 cells were stimulated with escalating doses of AICAr and brequinar. As shown in Fig. 4*B*, brequinar phenocopied the effects of AICAr on S-phase arrest in a dose-dependent manner. However, AICAr showed biphasic effects with maximal effects on the S-phase arrest at a concentration (0.2 mm) that is lower than the concentration that we used in our previous studies (13). The similar biphasic effects of two doses of AICAr on the level of orotate and the rate of cell growth have been previously described in fibroblasts, but the mechanism has not been elucidated (18). When applied at suboptimal concentrations, AICAr (0.1 mm) and brequinar (0.1 μm) exerted a synergistic effect on the S-phase arrest. Maximal effects were observed by 0.2 mm AICAr and 0.5 mm brequinar, and no further increase was observed by the addition of their combinations. Moreover, not only the effects of 0.5 mm AICAr on S-phase arrest were less pronounced than the effects of 0.2 mm AICAr, but AICAr at this concentration significantly reduced brequinar-mediated effects. As shown in Fig. 4*C*, the effects of increasing doses of AICAr, brequinar, and their combinations on the expression of differentiation markers, especially CD11b, mirrored the effects of agents on S-phase arrest. Therefore, we concluded that AICAr had biphasic effects on S-phase arrest and differentiation and that the low dose of AICAr (0.2 mm) shares the same mechanism of action as brequinar.

### AICAr inhibits UMP synthesis at a step downstream of DHODH

**Figure 5.**
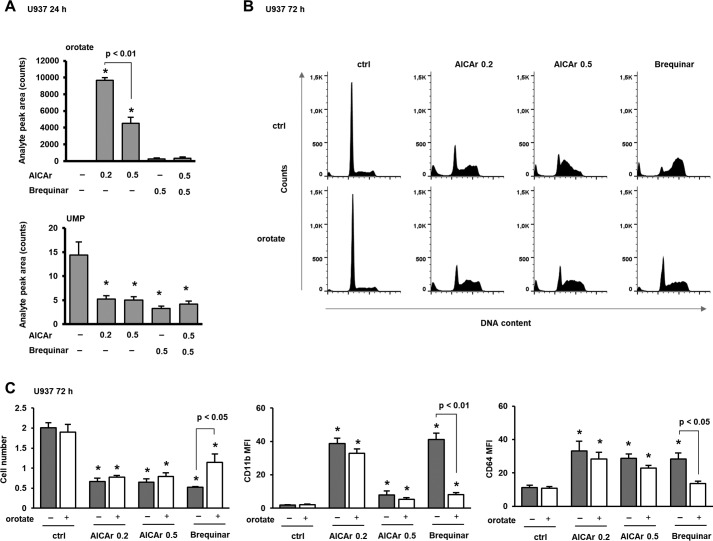
**AICAr increases the level of orotate and decreases the level of UMP; exogenous orotate prevents brequinar effects and has no effects on AICAr.** *A*, U937 cells were incubated with AICAr (0.2 or 0.5 mm) or brequinar (0.5 μm) for 24 h. The levels of orotate and UMP were determined as described under “Experimental procedures.” *B* and *C*, U937 cells were incubated with AICAr (0.2 or 0.5 mm) or brequinar (0.5 μm) for 72 h. Orotates (0.3 mm) were added 5–15 min after agents, and control cells were treated with vehicle (DMSO). *B*, representative histograms of propidium-labeled cells from three independent experiments analyzed by flow cytometry. *C*, the number of viable cells and the expression of differentiation markers were determined as described under “Experimental procedures.” Results are mean ± S.E. (*error bars*) of at least three independent experiments. *, *p* < 0.05 compared with control (*ctrl*).

If AICAr inhibits uridine synthesis at a step downstream of brequinar, we would predict that AICAr would reduce the intracellular concentration of UMP and increase that of orotate. Therefore, we next measured the level of UMP and orotate in cells treated with two different concentrations of AICAr and brequinar for 24 h. Consistent with our prediction, both AICAr and brequinar decreased the level of UMP, but only AICAr significantly increased the level of orotate, and the increase was significantly higher in cells treated with a lower concentration of AICAr. Brequinar reduced the level of UMP and abolished an increase in the level of orotate in cells treated with 0.5 mm AICAr (Fig. 5*A*).

In addition, we tested whether exogenously added orotate prevents AICAr- and brequinar-induced differentiation and S-phase arrest. When applied at the concentration (0.3 mm) that had no effect on the number of viable cells, orotate inhibited brequinar- but not AICAr-induced cycle arrest and differentiation (Fig. 5, *B* and *C*). These results indicate that AICAr inhibits pyrimidine synthesis at a step downstream of DHODH.

### AICAr induces differentiation through activation of DNA damage checkpoint kinase Chk1 via pyrimidine depletion

**Figure 6.**
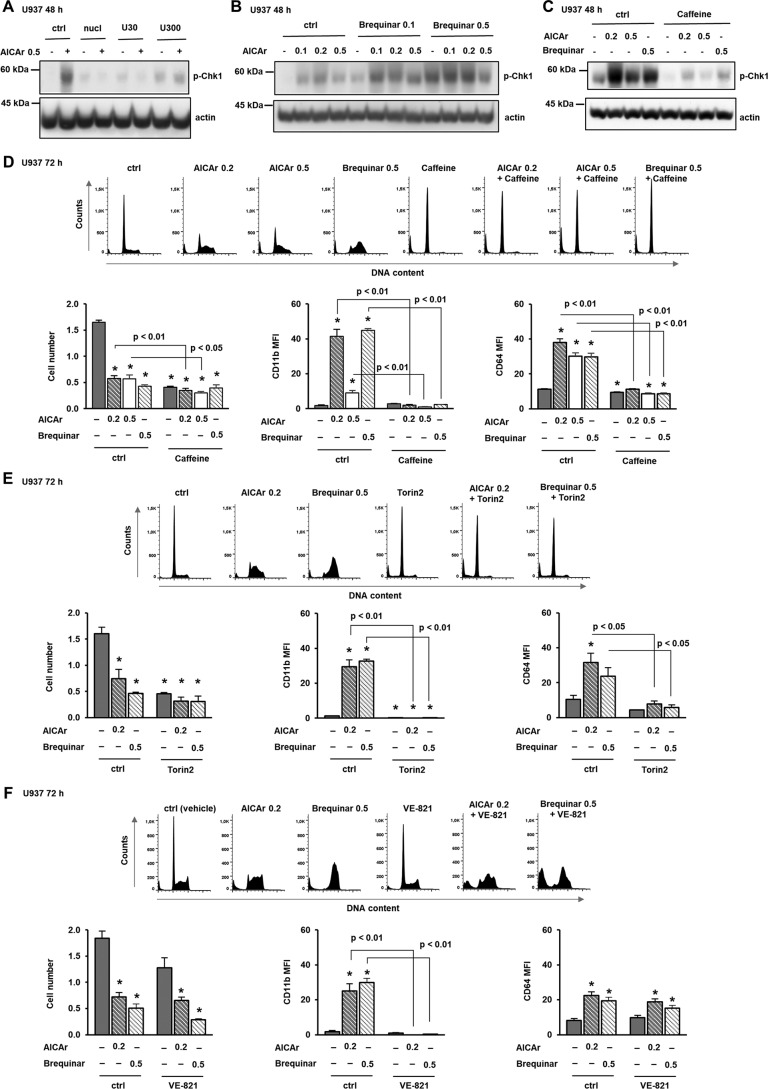
**AICAr activates Chk1, and pharmacological inhibition of ATR/Chk1 prevents differentiation and cell cycle arrest.** *A–C*, U937 cells were grown in the presence of AICAr (0.5 mm), nucleosides, and uridine (30 and 300 μm) (*A*); increasing concentrations of AICAr (0.1, 0.2, or 0.5 mm), brequinar (0.1 or 0.5 μm), and their combinations (*B*); or AICAr (0.2 and 0.5 mm) and brequinar (0.5 μm) with caffeine (4 mm) added 5–15 min prior to the addition of agents (*C*). Total cell lysates were isolated after 48 h and analyzed by Western blotting for the level of Ser-345–phosphorylated Chk1. Representative immunoblots from three independent experiments are shown. *D–F*, U937 cells were incubated with AICAr (0.2 and 0.5 mm) and brequinar (0.5 μm) for 72 h. Caffeine (4 mm) (*D*), Torin2 (100 nm) (*E*), and VE-821 (2 μm) (*F*) were added 30 min before the addition of agents. Representative histograms of propidium-labeled cells are shown, and the number of viable cells and the expression of differentiation markers were determined as described under “Experimental procedures.” Results are mean ± S.E. (*error bars*) of at least three independent experiments. *, *p* < 0.05 compared with control (*ctrl*).

Depletion of nucleotide pools is known to activate the DNA damage signaling pathway through activation of the ataxia telangiectasia and Rad3-related (ATR) kinase-mediated checkpoint in S phase (16). Chk1 acts downstream of ATR and regulates cell cycle progression (19). To determine whether AICAr-induced cell cycle arrest was associated with activation of the ATR/Chk1 DNA damage response, we determined the level of Chk1 phosphorylation on Ser-345 in cells treated with AICAr for 48 h. As shown in Fig. 6*A*, the level of Ser-345–phosphorylated Chk1 was increased in AICAr-treated cells, and the addition of either mixture of nucleosides or uridine abolished the phosphorylation of Chk1, demonstrating that Chk1 activation is caused by pyrimidine depletion.

To further test for the role of Chk1 activation in the effects of brequinar and AICAr, the level of Ser-345 p-Chk1 was assessed in lysates of cells treated with increasing doses of both drugs for 48 h. The increase in the level of Ser-345 p-Chk1 in response to all concentrations of AICAR, brequinar, and their combinations (Fig. 6*B*) followed the same pattern as the expression of differentiation markers and S-phase arrest (Fig. 4).

To further clarify the role of Chk1 activation in AICAr-mediated effects, U937 cells were pretreated with caffeine, an inhibitor of ATR (19). The presence of caffeine at a concentration that decreased Chk1 phosphorylation (Fig. 6*C*) prevented the arrest in S phase of the cell cycle and reduced the expression of differentiation markers in both AICAr- and brequinar-treated cells (Fig. 6*D*). Then we tested the effects of Torin2, an ATP-competitive inhibitor of mTOR, ATM, and ATR (20). As shown in Fig. 6*E*, Torin2 induced a G_0_/G_1_ arrest and completely prevented the effects of AICAr and brequinar on cell cycle progression and the expression of differentiation markers. VE-821 has been recently used as a selective inhibitor of ATR in U937 cells (21). Pretreatment with VE-821 at the concentration (2 μm) that has no single-agent activity consistently decreased the percentage of viable cells treated with both AICAr and brequinar, partially relieved their cell cycle block, and completely inhibited the increase in the expression of CD11b (Fig. 6*F*). These results demonstrate that AICAr and brequinar share the common pathway of DNA damage response that is necessary for differentiation.

**Figure 7.**
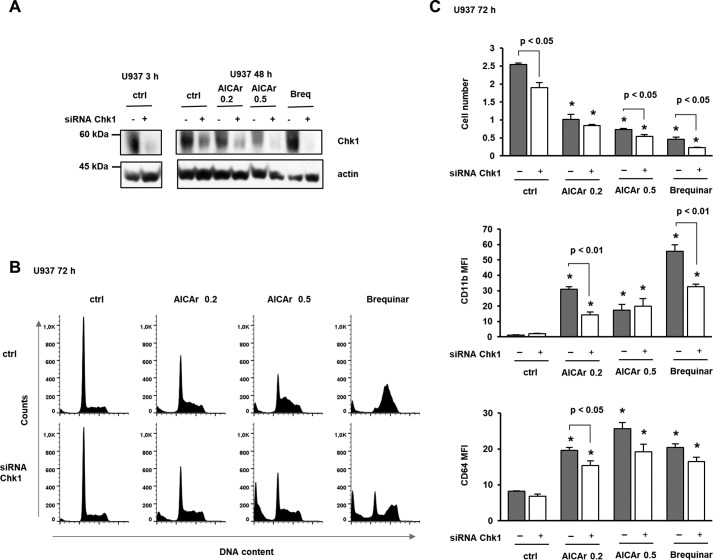
**Down-regulation of Chk1 reduced the effects of AICAr and brequinar on the expression of differentiation markers and S-phase arrest.** U937 cells were transfected with siRNA against Chk1 (mixture of four sequences), and respective nontargeting siRNA was used as a negative control. AICAr (0.2 or 0.5 mm) and brequinar (*Breq*) (0.5 μm) were added 24 h after transfection. *A*, total cell lysates were isolated 3 or 48 h after the addition of agents and analyzed by Western blotting for the level of Chk1. Representative immunoblots from three independent experiments are shown. *B*, representative histograms of propidium-labeled cells. *C*, the number of viable cells and the expression of differentiation markers were determined as described under “Experimental procedures.” Results are mean ± S.E. (*error bars*) of three independent experiments. *, *p* < 0.05 compared with control (*ctrl*).

To determine whether genetic down-regulation of Chk1 abrogates AICAr- and brequinar-induced differentiation, the cells were transfected with small interfering RNA (siRNA) targeting Chk1. As shown in Fig. 7, transfection decreased the level of Chk1 and significantly reduced the effects of AICAr and brequinar on the expression of differentiation markers and cell cycle arrest. Together, these results confirm that AICAr-mediated differentiation of U937 cells is mediated by activation of DNA damage checkpoint kinase Chk1 induced by pyrimidine depletion.

### AICAr induces cell cycle arrest and differentiation in other monocytic cell lines, and these effects are abolished by uridine

**Figure 8.**
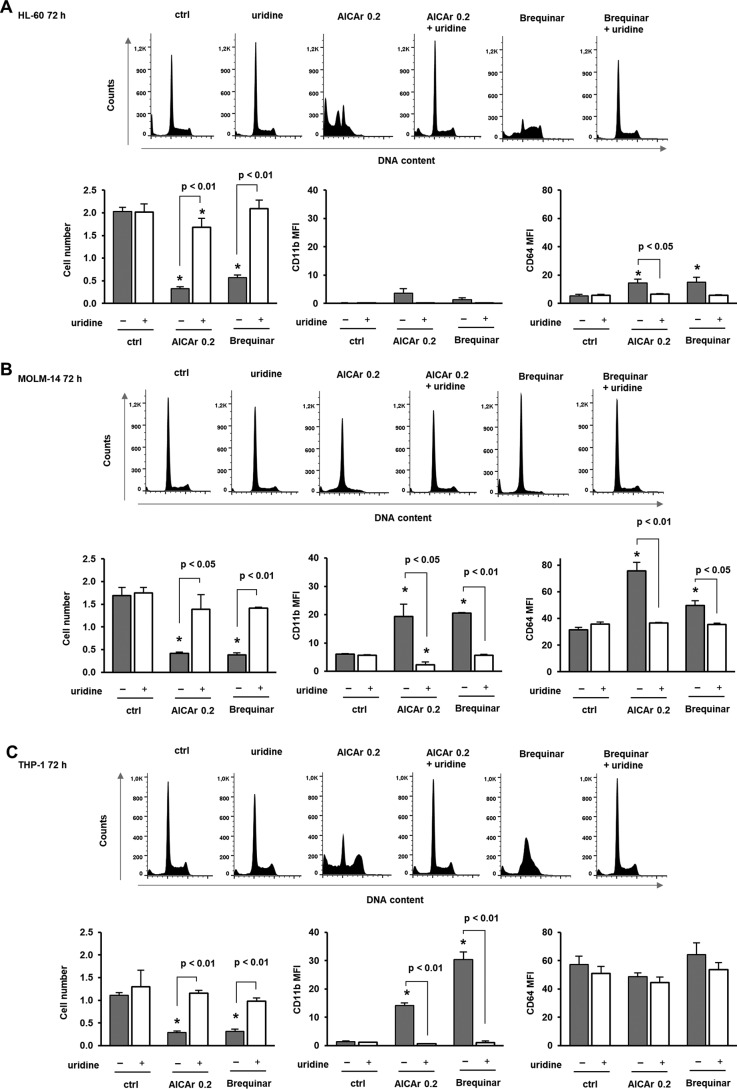
**AICAr induces cell cycle arrest and differentiation in other monocytic cell lines, and these effects are abolished by uridine.** Myeloblastic HL-60 (*A*), monocytic MOLM-14 (*B*), and THP-1 (*C*) cells were incubated with AICAr (0.2 mm) or brequinar (0.5 μm) and uridine (300 μm) for 72 h. Representative histograms of propidium-labeled cells analyzed by flow cytometry are shown. The number of viable cells and the expression of differentiation markers were determined as described under “Experimental procedures.” Results are mean ± S.E. (*error bars*) of at least three independent experiments. *, *p* < 0.05 compared with control (*ctrl*).

Myeloblastic HL-60 and monocytic MOLM-14 and THP-1 cells were next treated in the presence of AICAr to test whether similar effects could be observed in leukemia cell lines other than U937 cells (Fig. 8). In the myeloblastic HL-60 cell line, AICAr induced apoptosis but had no significant effects on CD11b expression as described previously (13). However, in monocytic MOLM-14 and THP-1 cell lines, both AICAr and brequinar prevented S-phase progression and increased the expression of differentiation markers. Furthermore, the addition of uridine completely abrogated the effects of AICAr and brequinar in both cell lines. These results prove that AICAr-induced differentiation via pyrimidine depletion occurs in multiple human leukemia cell lines.

## Discussion

Our results that AICAr-mediated expression of differentiation markers in myeloid leukemia cells can be abolished by the addition of uridine or a mixture of nucleosides are in line with previous studies demonstrating the importance of the pyrimidine biosynthetic pathway as a regulator of myeloid differentiation. An increase in the level of orotate and a decrease in the level of UMP in cells treated with AICAr suggest that AICAr inhibits UMP synthesis, a step downstream of DHODH. In our study, low doses of AICAr and brequinar exerted synergistic effects, and uridine abolished the effects of both drugs, further suggesting that the effects are due to the inhibition of UMP synthesis and not the accumulation of substrates. Therefore, AICAr can be placed on the list of pharmacological inhibitors that increase the expression of differentiation markers by inhibiting a particular step in pyrimidine synthesis, which includes DHODH inhibitors brequinar and leflunomide and OMP decarboxylase inhibitor pyrazofurin (3).

Because of structural similarities with adenosine, AICAr was first developed to block adenosine reuptake in ischemic heart disease (22), then used extensively as an allosteric activator of AMPK (12), and finally found to act as an “exercise in a pill” in sedentary mice (23). The most recent interest in another possible use of AICAr was initiated by studies reporting antiproliferative effects of metformin and other AMPK agonists in cancer (24). In AML cells, proapoptotic effects of metformin were independent of AMPK and associated with an increase in glycolysis (15). Our previous study showed that both drugs reduced the number of viable cells to a similar level, but no differentiation was observed in cells treated with metformin, and AMPK knockdown had no effect on AICAr-mediated differentiation (13). Consistent with a mechanism that does not involve AMPK activation and differs from the proapoptotic signal of metformin, AICAr-treated cells in our study did not exhibit increased glucose consumption.

In hematological malignancies, cytotoxic effects of AICAr were reported in lymphocytic leukemia, lymphoma, and chronic myeloid leukemia (25–27). In chronic myeloid leukemia, AMPK-independent cell death induced by AICAr involved autophagy (9), but our recent study showed that, although both ATRA and AICAr increased autophagy flux in parallel with differentiation, the effects of AICAr were not abolished by down-regulation of key proteins of the classical autophagy pathway (28). In lymphoid malignancies in which AICAr is known to induce antiproliferative effects, similar effects of AICAr on nucleotide synthesis have been reported. In lymphoblastic leukemia cell lines, an inhibition of UMP synthase was proposed as the mechanism responsible for the AICAr-mediated increase in the levels of orotate, dihydroorotate, and carbamoyl aspartate, and these effects were not mimicked by the selective AMPK activator A-769662 (29). Similar AMPK-independent effects of AICAr on UMP synthase inhibition have been observed in multiple myeloma cells (17). In both cell types, uridine decreased AICAr-induced apoptosis (17, 30), and a decrease in the level of 5-phosphoribosyl-1-pyrophosphate (PRPP) was proposed as the mechanism of UMP synthase inhibition (17). However, a decrease in PRPP alone cannot explain the unusual biphasic effect on cell growth and orotate levels in the model of fibroblasts because the level of PRPP remained low at all concentrations of AICAr. An important difference observed in fibroblasts was the fall in purine nucleotide concentration from supranormal to subnormal levels at a high concentration of AICAr (18), and there is a possibility that the inhibitory effects of a high dose of AICAr on brequinar-mediated differentiation in our model may be due to a decrease in purine nucleotides.

No data regarding the role of pyrimidine metabolism have been reported in models in which AICAr exerted differentiative effects, including astroglial differentiation of neural stem cells (31), erythroid differentiation of embryonic stem cells (32), and mineralization of osteoblastic MC3T3 cells (33). However, pyrimidine metabolism was one of three metabolic pathways, along with purine and folate pathways, that were significantly enriched in the core list of genes in AML cell lines treated with common differentiation inducers, like vitamin D or ATRA. Perturbation of the folate pathway by the knockdown of methylenetetrahydrofolate dehydrogenase–cyclohydrolase 2 (MTHFD2) inhibited growth and increased the level of CD11b in U937 cells (2). The level of endogenous AICAR upon knockdown of MTHFD2 in U937 cells has not been investigated, but the strongest metabolic change observed upon MTHFD knockdown in a different cell model was a dramatic elevation in the level of AICAR (34). These reports combined with our results suggest that an increase in the cellular level of AICAR, either from an endogenous source or added extracellularly, may induce differentiation of monocytic U937 cells.

What is the link between the lack of pyrimidine nucleotides and differentiation of leukemia cells? We show that both AICAr and DHODH inhibitor brequinar (3) induced Chk1 activation and subsequent S-phase arrest and that both pharmacologic and genetic inactivation of Chk1 abrogates both differentiation and S-phase delay. We therefore propose that activation of the DNA damage pathway is a critical mediator of AML differentiation in response to pyrimidine depletion. There are several possible downstream targets of activated Chk1 that have been associated with myeloid differentiation. In FLT3-ITD AML cells, both inhibition of CDK1 (35) and knockdown of Cdc25 (36) have been reported to promote differentiation as judged by CD11b expression, morphological changes, and phosphorylation of CCAAT-enhancer–binding protein α, a maturation-promoting transcription factor. Another potential mediator is p21, which was found to be necessary for DNA damage–induced cell cycle exit and differentiation of cells transformed with MLL-AF9 (37). In addition, retroviral transduction of p21 induced myeloid differentiation by lengthening the cell cycle and consequent accumulation of the lineage-determining PU.1 transcription factor, which favors macrophage differentiation (38). Differentiation induced by activation of DNA damage is not limited to hematologic cells as similar effects have been described in other cell types, including embryonic stem cells (39) and melanocytic stem cells (40).

In summary, we conclude that AICAr-mediated differentiation of monocytic U937 cells and S-phase arrest depend on pyrimidine synthesis and Chk1 activation. Further studies are needed to define what makes myeloid leukemia cells more sensitive to differentiative *versus* proapoptotic effects of AICAr and to determine whether AICAr induces differentiation in AML blasts from patients.

## Experimental procedures

### Reagents

AICAR (A9978), caffeine (C0750), and uridine (U3750) were purchased from Sigma and dissolved in sterile water to stock concentrations of 100, 80, and 30 mm, respectively. Torin2 (SML1224) and VE-821 (SML1415) were purchased from Sigma and dissolved in DMSO to a stock concentration of 10 mm. ATRA (554720) was purchased from Calbiochem and dissolved in DMSO to a stock concentration of 1 mm. 1,1-Dimethylbiguanide hydrochloride (metformin; D150959) was obtained from Sigma and dissolved in RPMI 1640 medium to a stock concentration of 1 m. Uridine 5-monophophate (U6375) and EmbryoMax Nucleosides (ES-008-D) were obtained from Sigma. Orotate (O2750) were obtained from Sigma and dissolved in DMSO to a stock concentration of 0.1 m. Anti-CD11b-FITC (IM0530), anti-CD64-FITC (IM1604), and FITC-conjugated mouse immunoglobulin G1 (IgG1) (IM0639) were purchased from Immunotech Beckman Coulter (Marseilles, France). Human TruStain FcX^TM^ (422302) was purchased from BioLegend (San Diego, CA). Cell lysis buffer (catalog number 9803), antibodies against Ser-345 p-Chk1 (catalog number 2348), Chk1 (catalog number 2360), anti-rabbit IgG (catalog number 7074), and anti-mouse IgG (catalog number 7076) conjugated to horseradish peroxidase were purchased from Cell Signaling Technology (Beverly, MA). Enhanced chemiluminescence (ECL) substrate was obtained from Thermo Fisher Scientific (Waltham, MA), and Bradford reagent (B6916) was from Sigma. Neon Transfection System 100 μl kit (catalog number MPK10096) was purchased from Invitrogen. ON-TARGETplus SMARTpool Human CHK1 (L-003255-00-0005) siRNA and negative control (D-001810-10) siRNA were obtained from Dharmacon (Lafayette, CO). Monoclonal anti-β-actin antibody (A5441), propidium iodide (PI), RNase A, Igepal, color markers, bovine serum albumin (BSA), Triton X-100, sodium dodecyl sulfate (SDS), leupeptin, and phenylmethylsulfonyl fluoride were purchased from Sigma. RPMI 1640 medium, fetal bovine serum, penicillin/streptomycin, and l-glutamine were obtained from Gibco/Invitrogen.

### Cell culture

U937 and HL-60 cells (ECACC numbers 88112501 and 98070106) were purchased from the European Collection of Authenticated Cell Cultures (Porton, Salisbury, UK). MOLM-14 and THP-1 (DSMZ numbers ACC 777 and ACC 16) were purchased from Deutsche Sammlung von Mikroorganismen und Zellkulturen GmbH (Braunschweig, Germany). Cell lines were expanded and frozen at early passages and used for the experiments within 10 weeks after being thawed from frozen stocks. The cells were maintained in RPMI 1640 medium supplemented with 10% heat-inactivated fetal bovine serum, 2 mm l-glutamine, 50 IU/ml penicillin, and 50 μg/ml streptomycin at 37 °C in a humidified atmosphere containing 5% CO_2_.

For the experiments, cells were harvested, resuspended in fresh medium, and seeded at a concentration of 0.2 × 10^6^/ml in 6-well plates or 0.3 × 10^6^/ml in 25-cm^2^ flasks. The cells were incubated in the presence of agents for various time intervals as indicated in the figure legends.

### Metabolic assays

Glucose (MAK263) and lactate (MAK065) assay kits were purchased from Sigma. The concentrations of metabolites in cell culture media were measured according to the manufacturer's instructions in supernatants obtained after sample centrifugation, and the values were standardized to the number of cells in the sample. The colorimetric measurements were performed in three independent experiments in triplicate using a Bio-Rad 680 microplate reader at 595 and 450 nm, respectively.

### Flow cytometric analysis

The expression of surface markers CD11b and CD64 was measured by flow cytometric analysis as described previously (41). Briefly, an aliquot of cells was washed and incubated with FITC-conjugated antibodies against CD11b and CD64 or with their isotypic control for 20 min in the dark. MOLM-14 and THP-1 cells were previously incubated with 2.5% Fc-blocking buffer for 10 min in the dark. After incubation, cells were washed and analyzed using the FACSCalibur System (BD Biosciences) and CellQuest software (BD Biosciences). Live cells were gated based upon forward and side scatter patterns, and a total of 15,000 events were collected for each marker from this gated area. To determine the mean fluorescence intensity (MFI) of the sample, MFI levels of isotypic controls were subtracted from MFI levels of the cells stained with CD-specific antibodies.

For cell cycle analysis, an aliquot of cells was washed and stained with PI solution (50 μg/ml PI, 10 mm Tris, pH 8.0, 10 mm NaCl, 10 μg/ml RNase A, 0.1% Igepal) and incubated at +4 °C for 20 min. DNA analysis was performed by collecting 10,000 events for each sample gated to eliminate aggregates and cell debris using the FACSCalibur System. The percentage of cells in the particular phase of the cell cycle was determined using ModFit software (BD Biosciences), and representative histograms were created using FlowJo (FlowJo LLC, Ashland, OR).

### Western blot analysis

Isolation of total cell lysates was performed as described previously (42). Briefly, cells were collected and washed in ice-cold PBS. The cell pellet was resuspended in 1× cell lysis buffer supplemented with 1 mm phenylmethylsulfonyl fluoride and 1 μm microcystin and incubated on ice for 10 min. To further enhance cell lysis, cells were disrupted by seven passages through a 23-gauge needle and incubated on ice for an additional 10 min. After centrifugation at 14,000 × *g* at 4 °C for 10 min, supernatants were collected and stored at −80 °C. The protein concentration of each sample was determined colorimetrically using Bradford reagent and an Eppendorf Biophotometer Plus at 595 nm.

For immunoblotting, equal amounts of proteins (50 μg) per well were loaded on an 8% SDS-polyacrylamide gel. Protein electrophoresis was performed using a Bio-Rad Mini-Protean system at a constant voltage of 100 V, and wet transfer to nitrocellulose membrane was carried out using a Bio-Rad Mini Trans-Blot system at a constant voltage of 7 V overnight. After transfer, the membrane was blocked in TBS-Tween buffer (25 mm Tris, 150 mm NaCl, 0.1% (v/v) Tween 20) containing 5% (w/v) nonfat dried milk for 30 min and incubated with primary antibodies diluted in TBS-Tween buffer containing 5% (w/v) BSA (1:20,000 for anti-actin antibody; 1:1000 for all other antibodies) at 4 °C overnight. The next day, membranes were washed three times in TBS-Tween buffer, incubated with secondary antibodies at room temperature for 2 h, and visualized using the ECL kit according to the manufacturer's instruction. Signal was detected either on X-ray film or using a ChemiDoc^TM^ XRS+ system (Bio-Rad).

### siRNA transfection

Transfection with CHK1 siRNA and corresponding control was performed using the Neon transfection system (Invitrogen) as described previously (28). Briefly, cells were washed in PBS and resuspended in transfection buffer at a concentration of 23 × 10^6^/ml. siRNA solutions were added so their volume did not exceed 14% of total volume. Electroporation was performed in 100-μl Neon tips using a single pulse at a voltage of 1050 V and pulse width of 50 ms. After electroporation, cells were incubated in medium without antibiotics for at least 16 h at 37 °C. The final concentration of siRNA against CHK1 was 130 nm. The next day, transfected cells were resuspended in fresh medium and plated in 6-well plates for cell cycle and differentiation experiments. An aliquot of cells was used for the preparation of total cell lysates, and the level of down-modulated proteins was determined by Western blot analysis.

### Metabolomic analysis by LC/MS/MS

Cells were incubated at a concentration of 0.25 × 10^6^/ml in the presence of drugs for 24 h. At the end of incubation, cells were lysed with ice-cold 80% methanol, vortexed, and stored at −20 °C. Stock standard solutions of UMP and orotate were prepared at a concentration of 0.1 mg/ml in 0.1% formic acid (v/v), methanol (50:50) and stored at −20 °C.

The standards and samples were analyzed using an Agilent 1290 Infinity LC coupled to an Agilent 6460 triple-quadrupole MS/MS with Jet Streaming technology and electrospray ionization (ESI) using Agilent MassHunter software (B.07.00) at BioCentre Zagreb. Separation was performed using a Synergy Polar-RP LC column (4 μm; 150 × 4.6 mm) heated to 40 °C in a column oven. The flow rate was 0.3 ml/min with mobile phase A (0.1% formic acid) and mobile phase B (100% methanol). The gradient was formed as follows: initial eluent, 90% A, 10% B; 20% A, 80% B at 7 min and held under these conditions for 1.5 min before returning to 90% A, 10% B. The concentrations of 90% A and 10% B were maintained up to 15 min. Data acquisition was performed in MRM mode with positive and negative ESI using one principal MRM transition for quantitation and one additional transition as a qualifier for each analyte (Table 1). MS source parameters were set as follows: gas temperature, 250 °C; gas flow, 7 liters/min; nebulizer, 25 p.s.i.; sheath gas heater, 350 °C; sheath gas flow, 10 liter/min; capillary voltage, 3500 V; and charging voltage, 1500 V.

**Table 1:** **Transition list**

Compound name	Precursor ion	ESI polarity	Fragmentor	Fragment ions	Collision energy	Dwell (ms)	Cell acceleration
	*m/z*		*V*		*V*		*V*
Orotate	157.1	Positive	90	110.8*^a^*	14	15	7
Orotate	157.1	Positive	90	68.1	24	15	7
UMP	323.5	Negative	130	150.8*^a^*	16	15	7
UMP	323.5	Negative	130	97.0	18	15	7

*^a^* MRM transition used for relative quantitation.

### Statistical analysis

Statistical analysis was performed using Microsoft Excel and GraphPad Prism 6 (GraphPad Software) to calculate the *p* values for unpaired *t* test and analysis of variance with post hoc Tukey test. The results were considered to be statistically significant if *p* was <0.05. Data are presented as mean ± S.E. of at least three independent experiments.

## Author contributions

V. D., J. A. S., A. B., and D. V. conceptualization; V. D., B. T., and D. V. data curation; V. D., B. T., I. K., J. A. S., A. B., and D. V. formal analysis; V. D., J. A. S., A. B., and D. V. validation; V. D., B. T., I. K., and D. V. investigation; V. D., B. T., I. K., and D. V. visualization; V. D., B. T., I. K., and D. V. methodology; V. D., A. B., and D. V. writing-original draft; V. D., B. T., I. K., J. A. S., A. B., and D. V. writing-review and editing; J. A. S., A. B., and D. V. funding acquisition; D. V. resources; D. V. supervision; D. V. project administration.

## Footnotes

2 The abbreviations used are:

ATRA: all-*trans*-retinoic acid
APL: acute promyelocytic leukemia
AML: acute myeloid leukemia
IDH: isocitrate dehydrogenase
MRM: multiple reaction monitoring
ESI: electrospray ionization
AICAr: 5-aminoimidazole-4-carboxamide ribonucleoside
AMPK: AMP-activated protein kinase
Chk1: checkpoint kinase 1
DHODH: dihydroorotate dehydrogenase
UMP: uridine monophosphate
AICAR: 5-aminoimidazole-4-carboxamide ribonucleotide
ATR: ataxia telangiectasia and Rad3-related
DHO: dihydroorotate
p-Chk1: phosphorylated Chk1
ATM: ataxia telangiectasia mutated
PRPP: 5-phosphoribosyl-1-pyrophosphate
MTHFD: methylenetetrahydrofolate dehydrogenase–cyclohydrolase
PI: propidium iodide
MFI: mean fluorescence intensity.
